# Passive sampling hypothesis did not shape microbial species–area relationships in open microcosm systems

**DOI:** 10.1002/ece3.9634

**Published:** 2022-12-15

**Authors:** Wei Deng, Yi‐Ting Cheng, Zheng‐Qiang Li, Fa‐Ping Zhou, Xiao‐Yan Yang, Wen Xiao

**Affiliations:** ^1^ Institute of Eastern ‐ Himalaya Biodiversity Research Dali University Dali Yunnan China; ^2^ The Provincial Innovation Team of Biodiversity Conservation and Utility of the Three Parallel Rivers Region Dali University Dali Yunnan China; ^3^ Collaborative Innovation Center for Biodiversity and Conservation in the Three Parallel Rivers Region of China Dali Yunnan China; ^4^ International Centre of Biodiversity and Primates Conservation Dali Yunnan China

**Keywords:** dispersal mechanisms, island biogeography, passive sampling hypothesis, sampling effects, species richness

## Abstract

The passive sampling hypothesis is one of the most important hypotheses used to explain the mechanism of species–area relationships (SAR) formation. This hypothesis has not yet been experimentally validated due to the confusion between passive sampling (a larger area may support more colonists when fully sampled) and sampling effects (more sampling effort will result in increased species richness when sampling is partial). In this study, we created an open microcosm system with homogeneous habitat, consistent total resources, and biodiversity background using Chinese paocai soup, a fermented vegetable, as a substrate. We made efforts to entirely exclude the influence of sampling effects and to exclusively obtain microorganisms from dispersal using microcosm and high‐throughput sequencing techniques. However, in this study, passive sampling based on dispersal failed to shape SAR, and community differences were predominantly caused by species replacement, with only minor contributions from richness differences. Ecological processes including extinction and competitive exclusion, as well as underlying factors like temporal scales and the small island effects, are very likely to have been involved in the studied system. To elucidate the mechanism of SAR development, future studies should design experiments to validate the involvement of dispersal independently.

## Graphical abstract

Although the passive sampling hypothesis was not established, the experimental design of this study completely removed the role of pure sampling effects and returned to the essence of the passive sampling hypothesis of dispersal.

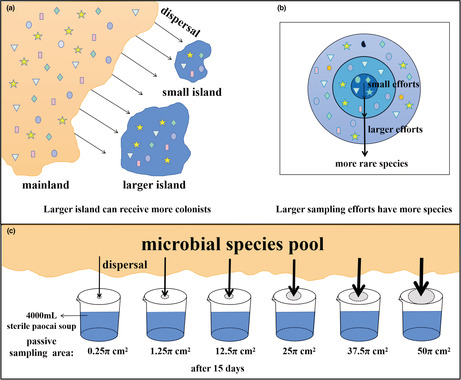

## 1 INTRODUCTION

The species–area relationships (SAR), whereby species richness increases with the area, is one of the few laws in ecology (Adler & Lauenroth, 2003; MacArthur et al., 1965; Rosindell & Chisholm, 2021). Although SAR research has become a hot topic in ecology today, some of the hypotheses used to explain its formation are still unclear (Gooriah & Chase, 2020; Gooriah et al., 2021; Ohyama et al., 2021; Tjørve et al., 2017).

Several commonly established hypotheses exist: (1) The area per se hypothesis, whereby larger areas tend to have lower rates of species extinction, and the equilibrium theory of island biogeography developed on this basis is also an important mechanism for explaining SAR (Ben‐Hur & Kadmon, 2020; Connor & McCoy, 2013; MacArthur et al., 1965; Ricklefs & Lovette, 1999; Williams et al., 1943); (2) The habitat diversity hypothesis contends that larger areas have more diverse habitats (Connor & McCoy, 2013); (3) the environmental filtering hypothesis contends that as abiotic selection intensifies, less adapted species on smaller islands are more likely to go extinct than those on larger islands (Chisholm et al., 2016; Liu et al., 2020); and (4) the passive sampling hypothesis contends that a larger area can accept more colonists (Ben‐Hur & Kadmon, 2020; Connor & McCoy, 2013). Among them, the passive sampling hypothesis is the most controversial hypothesis to explain the SAR mechanism, it is even considered to be just an invalid hypothesis (Coleman et al., 1982; Cam et al., 2002; Ouin et al., 2006).

**FIGURE 1:**
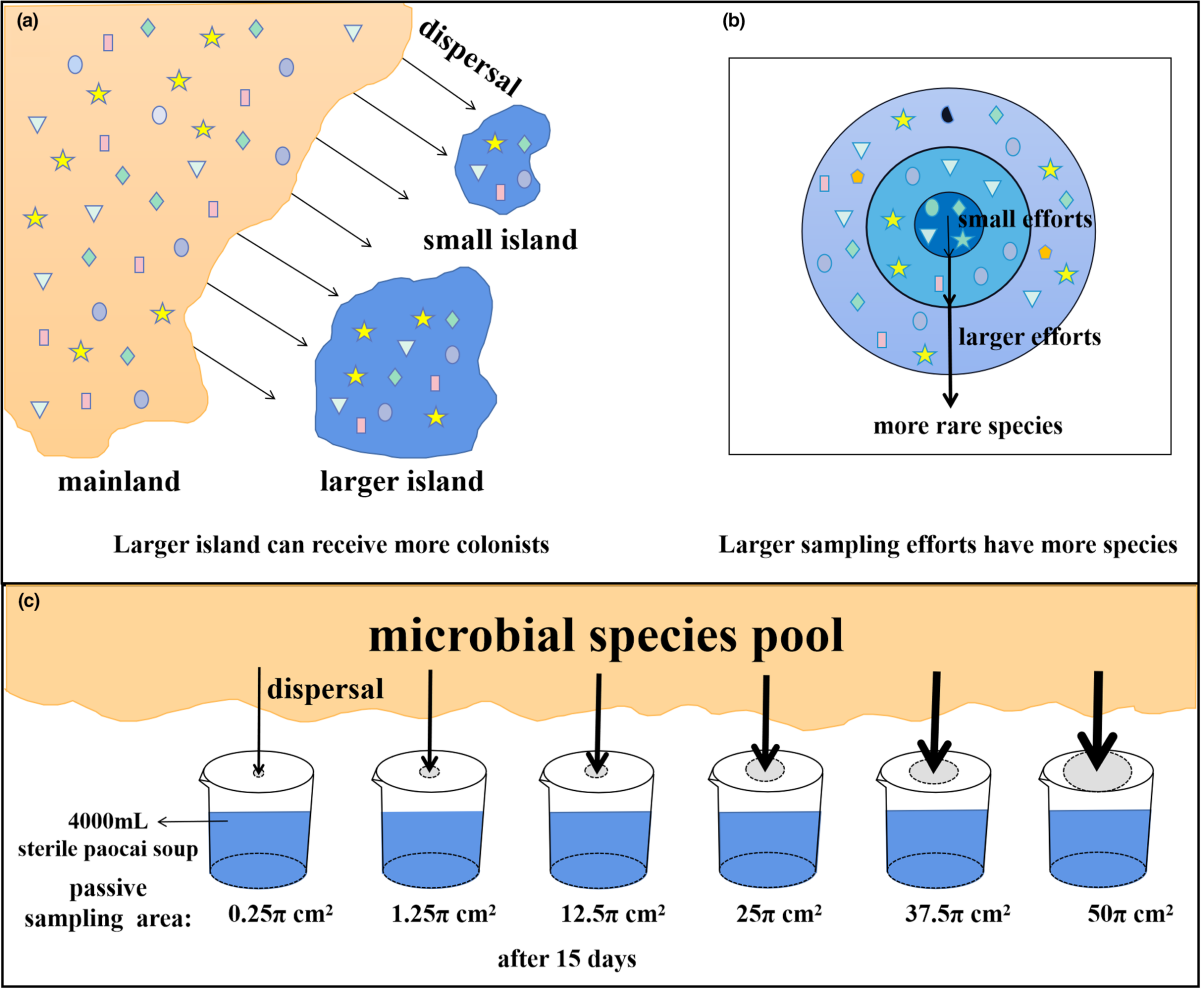
Schematic diagram of the hypothesis. (a): Schematic diagram of the passive sampling hypothesis. The arrows represent the dispersal process, the yellow areas represent the mainland species pool, the blue areas represent small and larger islands respectively, and the patterns on the areas represent different species. (b): Schematic diagram of nested sampling. The different colored areas represent sampling areas, the patterns on the areas represent different species, and the different thick and thin arrows represent species richness. (c): Passive sampling experimental design diagram. The yellow area at the top represents the pool of microbial species from which all the dispersal species come; the black arrows of different thicknesses represent the amount of dispersal that can be accepted for different passive sampling areas, generally, the larger the area, the more colonists be received; the blue area in the six equal‐sized beakers represents the 4000 mL of paocai soup added, The grey areas on the beaker represent the passive sampling areas, increasing from left to right.

The passive sampling hypothesis is one of the main theories used to explain how SARs emerge (Burns et al., 2009; Cam et al., 2002; Gooriah et al., 2021; Yamaura et al., 2016). This hypothesis outlines an island species–area relationships (ISAR), which is an independent systematic sampling used to forecast the colonists on islands by stochastic dispersal from the mainland. It was first put forth by Connor and McCoy in 1979 based on islands. According to experts, there is a sizable population of species on the mainland that disperse at random to islands, with larger islands acquiring more colonists (Coleman et al., 1982; Connor & McCoy, 1979; Connor & McCoy, 2013) (Figure 1a). According to these views, Passive sampling should be a distinct ecological process that emphasizes the reception of colonists from the species pool (Cam et al., 2002).

In subsequent research, the study of the passive sampling hypothesis is conducted only on the mainland (Kelly et al., 1989; Tangney et al., 1990). Species–area relationships have been ascribed to a sampling effect in continuous continental regions, i.e., phenomena in which the number of species observed grows with sampling effort when sampling is inadequate. Because the community usually contains many rare species which do not appear in all studied locations, even if their spatial distribution is random, they will only be gathered in larger areas (Wardle et al., 1999) (Figure 1b). Thus, while the sampling effect can generate monotonically increasing curves (Ouin et al., 2006; Preston et al., 1960), it is insufficient to generate the steeper slope of SAR curves (SARs). Such a curve is also not SARs (Cam et al., 2002; Ouin et al., 2006). In other words, in the absence of exhaustive detection of regional biodiversity, an increase in sampling area also implies an increase in sampling volume, which should result in species accumulation curves (SACs) shaped by sampling effects (Azovsky, 2011; Hui, 2008).

The difference between passive sampling and sampling effects is considerable. Passive sampling is the process of taking samples from a species pool as a result of random species dispersal (Burns et al., 2009; Guadagnin et al. 2009), whereas the sampling effect is the phenomenon in which species richness rises as sampling effort increases; it is just a product of sampling (a sampling artifact phenomenon) (Cam et al., 2002; Ouin et al., 2006; Rosenzweig, 1995). Such a sampling artifact phenomenon is challenging to eliminate from continental sampling, making it impossible to quantify the importance of ecological processes like dispersal. Some researchers contend that the only factor contributing to increased species richness is a sampling impact unrelated to ecological processes (Ben‐Hur & Kadmon, 2020; Bidwell et al., 2014). Some academics have also suggested that the correlation between species richness and sampling area may be the result of dispersal as well as sampling effects (Cam et al., 2002; Ouin et al., 2006; Storch et al., 2003). However, a large majority of people continue to believe that the sampling effects, not passive sampling, are what determine SAR (Ben‐Hur & Kadmon, 2020; Bidwell et al., 2014).

According to the equilibrium theory of island biogeography, the passive sampling hypothesis emphasizes that ISAR is shaped by trade‐offs among speciation, dispersal, and extinction, independent of sampling effects (MacArthur & Wilson, 2016). In fact, without eliminating sampling effects, SARs are not able to fully explain SAR and have much less theoretical and practical usefulness (Azovsky, 2011). The lack of a clear distinction between sampling effects and ecological processes in current SAR studies has complicated the investigation of the mechanisms behind SAR creation. Therefore, the original presumption that more species can spread into a greater area should be used to validate the passive sampling hypothesis. Experiments must clearly define the methods by which passive sampling affects SAR by concentrating on the role of dispersal after accounting for sampling effects and ecological processes (Gooriah & Chase, 2020; Gooriah et al., 2021).

Microbial microcosm systems are an excellent way to construct a system for studying species–area relationships (Deng et al., 2021; Deng et al., 2022). In this study, an open microcosm system was created utilizing equal parts of homogeneously mixed paocai soup from the same period and open beakers. High‐throughput sequencing of 16 S and ITS amplicon fragments from the system was then used to conduct SAR investigations. It posed to explore the microbial SAR shaping by passive sampling. We hypothesize that after the sampling effect is completely removed, the species richness will also increase significantly with the increase in passive sampling area, and SARs can be constructed.

## 2 METHODS

### 2.1 Experimental design

Microcosm systems were established using beakers with different opening sizes, and well‐mixed and sterilized paocai soup (Figure 1c). The same period of paocai soup in microbial microcosm research systems ensures a consistent starting point for microbial colonization, a well‐mixed paocai soup controls for the effects of habitat heterogeneity and biodiversity context, and equal amounts of paocai soup ensures a consistent total amount of resources in each microcosm. Additionally, the increasingly sophisticated high‐throughput sequencing technology provides the opportunity to eliminate sampling effects. To start, the experiment's beaker volume is just the right size to allow for thorough sampling. Secondly, to conduct our research at a finer amplified subsequence variants (ASVs) categorization level, we used high‐throughput sequencing technologies. This guarantees that our monitoring is rather thorough. By controlling for factors other than area to be consistent and only gradually increasing the beaker opening area while placing the entire microcosm in the open space for passive microbial sampling. The aforementioned experimental system was able to meet the requirements for testing the passive sampling hypothesis.

### 2.2 Preparation of paocai

We mixed well 35 kg of white radish (*Raphanus sativus*), 35 kg of cabbage (*Brassica oleracea*), 2 kg of pepper (*Capsicum frutescens*), 1 kg of ginger (*Zingiber officinale*), 1 kg of pepper (*Zanthoxylum bungeanum*), 2.5 kg rock sugar, and 210 kg cold boiling water (6% salt), and packed the mixture in paocai jars, and sealed for 7 days.

### 2.3 Handling of paocai soup

We filtered the paocai soup through sterile gauze to obtain paocai soup. The filtered paocai soup was sealed and precipitated for 12 h. The supernatant was left, the precipitate was removed, and the paocai soup was repeatedly filtered 2–3 times to obtain a homogeneous texture of paocai soup. The treated paocai soup was then sterilized to ensure a consistent background of microbial diversity and environmental heterogeneity.

### 2.4 Microbiological sampling

A total of six identical sized beakers (5000 ml) were used, each with an equal amount (4000 ml) of paocai soup. A hole punch was used to punch holes in the lids of the beakers and the passive sampling area was controlled by controlling the number of holes punched, resulting in passive sampling areas of 0.25π cm^2^, 1.25π cm^2^, 12.5π cm^2^, 25π cm^2^, 37.5π cm^2^, and 50π cm^2^, respectively (We using the na1‐6 represent the passive sampling areas from 0.25π to 50 cm^2^). After all microcosms were made, it was sealed with sterile newspaper and placed in an open space; next, the lids were opened at the same time for sampling and the timing started immediately (Figure 1c). The paocai soup was recovered after 15 days. The paocai soup in each microcosm was centrifuged for 15 min at 4°C, 8000 r/min, using a HC‐3018r high‐speed refrigerated centrifuge (Anhui USTC Zonkia Scientific Instruments Co., Ltd.). The supernatant was removed from the centrifuged paocai soup, leaving the precipitate immediately cryopreserved and sent to Wekemo Tech Group Co., Ltd. Shenzhen China.

### 2.5 Microbiological analysis

#### 2.5.1 DNA extraction and PCR amplification

Sequencing services were provided by Wekemo Tech Group Co., Ltd., Shenzhen China., using the E.Z.N.A.® soil kit (Omega Bio‐Tek, Norcross, GA, U.S.) to extract microbial DNA from the samples, using the NanoDrop 2000 kit to check DNA concentration and purity, and then using 1% agarose gel electrophoresis to check DNA extraction quality. Bacteria were amplified by PCR using primers 338 F (5′‐ACTCCTACGGGAGGCAGCAG‐3′) and 806 R (5′‐GGACTACHVGGGTWTCTAAT‐3′) targeting the V3‐V4 variable region of the 16 S ribosomal RNA (rRNA) gene (Wang et al., 2019). The fungi were amplified using ITS1‐1F‐F (5′‐CTTGGTCATTAGAGGAGTAA‐3′) and ITS‐1F‐R (5′‐GCTGCGTT CTTCATCGATGC‐3′) primers targeting the ITS1‐1F region of the ITS rDNA gene in the ribosome (Manter & Vivanco, 2007; Maryam et al., 2015; White et al., 1990).

#### 2.5.2 Amplification systems

PCR reactions were performed in triplicate on 20 μl mixture containing 4 μl of 5 × FastPfu buffer, 2 μl of 2.5 mM dNTPs, 0.8 μl of each primer (5 μM), 0.4 μl of FastPfu polymerase, and 10 ng of template DNA. The resulting PCR products were extracted from a 2% agarose gel and further purified using the AxyPrep DNA Gel Extraction kit (Axygen Biosciences, Union City, CA, USA) and quantified using QuantiFluor™‐ST (Promega, USA) according to the manufacturer's protocol. The PCR procedure for the 16 S rRNA gene was as follows: initial denaturation at 95°C for 3 min, 27 cycles (95°C for 30 s, annealing at 55°C for 30 s, and extension at 72°C for 45 s), and final extension at 72°C for 10 min. The PCR procedure for the ITS1‐1F region was as follows: initial denaturation at 98°C for 1 min, 30 cycles (denaturation at 98°C for 10 s, annealing at 50°C for 30 s, and extension at 72°C for 30 s), and final extension at 72°C for 10 min to achieve complete amplification of the target gene.

#### 2.5.3 Illumina NovaSeq sequencing

PCR products were extracted from 2% agarose gels, further purified using the AxyPrep DNA Gel Extraction kit (Axygen Biosciences, Union City, CA, USA), eluted in Tris–HCl, and detected by 2% agarose electrophoresis. Libraries were constructed using the Illumina TruSeqDNA PCR‐Free Library Preparation kit (Axygen Biosciences, Union City, CA, USA) and quantified using Quantiflur‐ST, and after all the above steps are qualified, the libraries were sequenced using NovaSeq 6000 PE250 sequencing (Bokulich et al., 2012; Qian et al., 2021).

#### 2.5.4 Data processing

Raw data FASTQ files were imported into the QIIME tool, and sequences from each sample were identified, quality filtered, trimmed, denoised, and merged using the QIIME2 dada2 plugin, and used to obtain feature tables of ASVs (Bolyen et al., 2019; Callahan et al., 2016; Nearing et al., 2018). Next, the QIIME2 feature‐classifier plugin was applied to match representative sequences of ASVs to the pre‐trained version 13_8 99% similarity GREEN GENES database (trimming the database to the region V3‐V4 based on 338F/806R primer pairs for bacteria) (DeSantis et al., 2006) and UNITE database (for fungi) to generate the taxonomy table (Bokulich et al., 2018). All contaminating mitochondria and chloroplast sequences were subsequently eliminated using the QIIME2 feature‐table plugin, excluding rare ASVs that accounted for less than 0.001% of the total sequences (Callahan et al., 2016; Marizzoni, 2020).

#### 2.5.5 Data analysis

All analyses involving the R language were done using R‐4.2.1 (https://cran.r‐project.org/src/base/R‐4.2.1). Using the vegan package in R (https://github.com/vegandevs/vegan; Oksanen et al., 2012), species rarefaction curves were plotted using the sequencing depth as the horizontal coordinate and ASV as the vertical coordinate. To show the diversity and structure of the microbial community, we used a hierarchical stacking diagram. And we draw the bar graph with the bar graph function in R, the passive sampling area is represented as the horizontal coordinate, and the paocai soup in the microcosm serves as the vertical coordinates to show the case of the microcosmic pH with different sampling areas. The number of ASVs for each sample was calculated from the feature table, and this number was the sample richness. SARs were plotted with the log‐transformed area as the horizontal coordinate and log‐transformed species richness as the vertical coordinate, using linearly fitted P‐values and slope values to determine whether there were significant species–area relationships. In addition, we calculated a linear correlation between log‐transformed species richness and microcosmic pH. Finally, β‐diversity partition was performed using the R language adespatial package (https://CRAN.R‐project.org/package=adespatial; Shen et al., 2020), and we used vegan, ggplot2 (https://ggplot2.tidyverse.org), and ggrepel packages (https://CRAN.R‐project.org/package=ggrepel) for redundancy analysis (RDA) (Forester et al., 2018).

## 3 RESULTS

**FIGURE 2:**
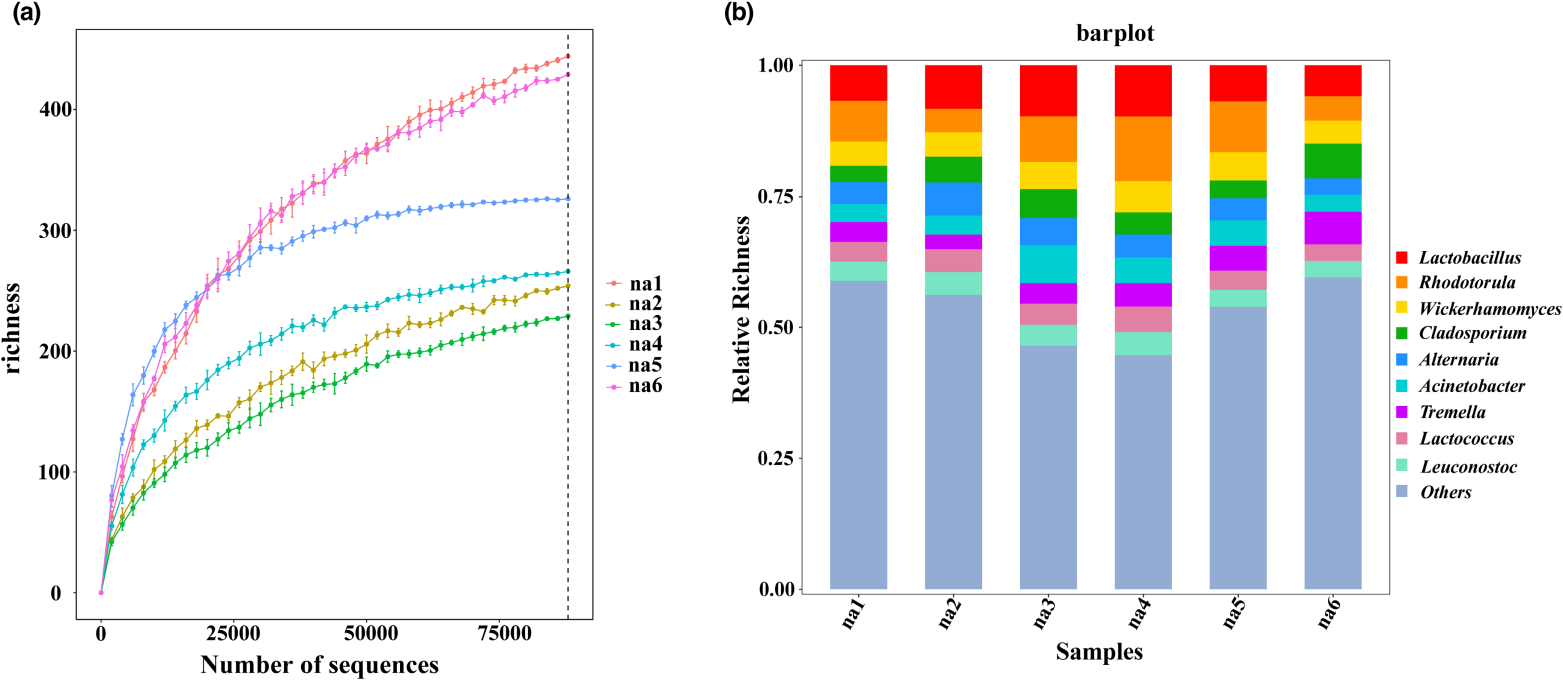
Sequencing signature map. (a) species rarefaction curves. The different colored curves represent different samples, which was used to assess the saturation of the sample size. The rarefaction curves obtained species richness at different sequencing depths by performing 2000 resampling at some sequencing depth. Each point on the curve represents the mean species richness at resampling, the error line is the standard error, and the dashed line indicates the saturation of the sample sequencing volume. (b) Stacking map at the genus level. The abscissa represents the different samples (We using the na1‐6 represent the passive sampling areas from 0.25π to 50 cm^2^), with the ordinate being the relative richness, where the colored blocks show the relative richness of the dominant genus in the microcosm.

As the depth of sequencing increased, the species rarefaction curves first increased and then rapidly leveled off (Figure 2a). A total of 527,742 microbial high‐throughput sequences were obtained from all samples, including 96,120 for bacteria and 431,622 for fungi. A total of 11,868 ASVs were obtained, of which 10,842 were bacterial and 1026 fungal. The dominant bacteria were *Lactobacillus*, *Acinetobacter*, *Lactococcus*, *and Leuconostoc*; the dominant fungi were *Rhodotorula*, *Wickerhamomyces*, *Tremella*, *Alternaria*, and *Cladosporium* (Figure 2b).

**FIGURE 3:**
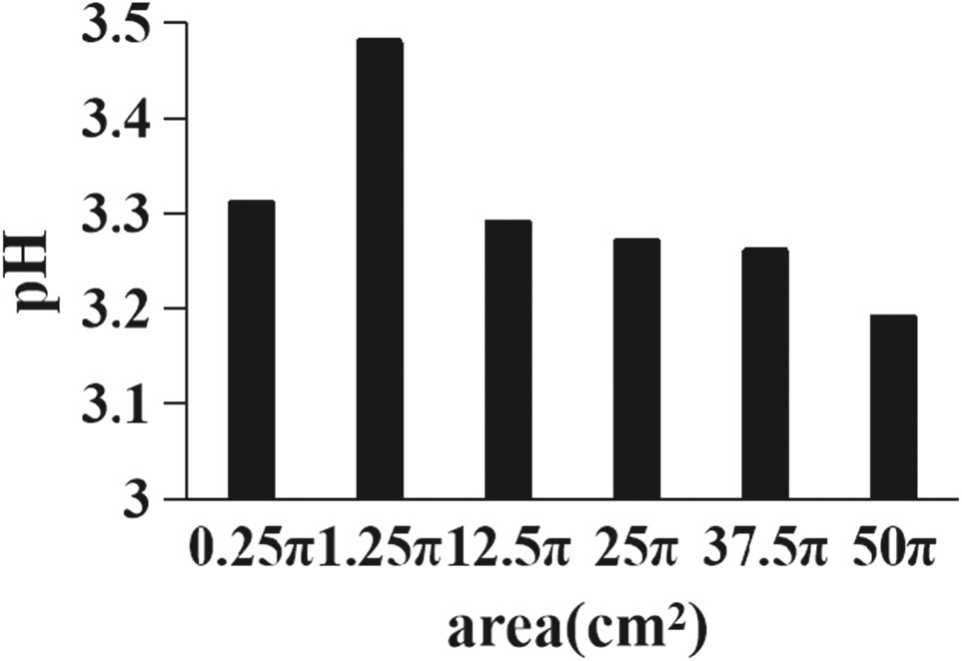
Variation in microcosmic pH by area. The horizontal coordinate represents the passive sampling area in cm^2^ and the vertical coordinate represents the pH of paocai soup in the microcosm.

The pH of paocai soup was 3.31, 3.48, 3.29, 3.27, 3.26, and 3.19 for the different microcosm systems. pH was greatest for the microcosm with an area of 1.25π cm^2^ and least for the microcosm with an area of 50π cm^2^ (Figure 3). Regression analysis showed no significant correlation between microbial richness and pH for paocai soup (*p* = .3359, *Slope* = −4.45). It showed no significant correlation between bacterial richness and pH (*p* = .3134, *Slope* = −5.114). And it showed no significant correlation between fungal richness and pH (*p* = .8944, *Slope* = .747).

**FIGURE 4:**
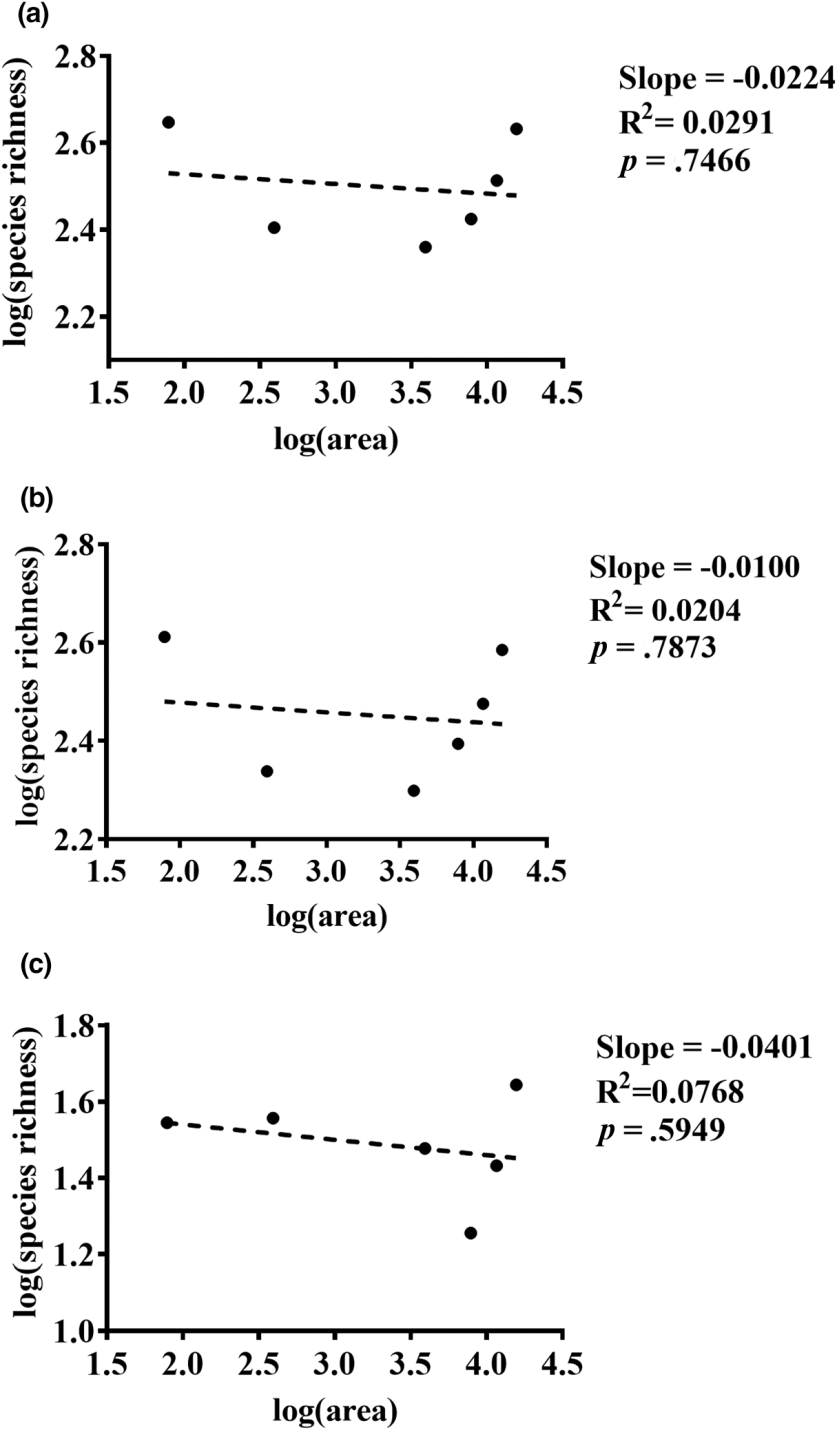
Species–area relationships curves. The horizontal coordinate represents the log‐transformed passive sampling area and the vertical coordinate represents the log‐transformed microbial species richness. (a) Variation in microbial richness with increasing area. (b): Variation in bacterial richness with the increasing area. (c): Variation in fungal richness with increasing area. Regression lines were calculated by linear models, with dashed lines indicating insignificant changes in species richness with the area.

There was no significant increase in species richness as the passive sampling area was changed. The overall microbial community did not show a significant SAR with increasing areas (Figure 4a: *Slope* = −.0224, *R* ^*2*^ = .0291, *p* = .7466). The bacterial community did not show a significant SAR as the area increased (Figure 4b: *Slope* = −.0100, *R* ^*2*^ = .0204, *p* = .7873). The fungal community did not show a significant SAR with increasing areas (*Slope* = −.0401, *R* ^*2*^ = .0768, *p* = .5949).

**FIGURE 5:**
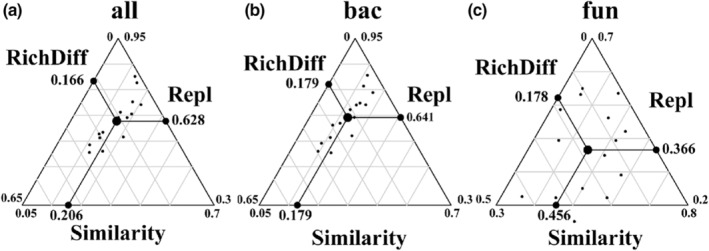
Microbial microcosm system β‐diversity partition diagram (a) all microbial β‐diversity partition in the microcosm system. (b) all bacterial β‐diversity partition in the microcosm system. (c) all fungal β‐diversity partition in the microcosm system. Triangular plots of β‐diversity partition result. Each black dot represents a pair of samples. Their positions were determined by a triplet of values from the species similarity (Similarity), species replacement (Repl), and species richness differences (RichDif); each triplet sums to 1. The large circular dot in each graph is the centroid of the points; the larger black dots represent the mean values of the Similarity, Repl, and RichDif.

To clarify the effect of different passive sampling areas on differences in microbial community composition in paocai soup, the β‐diversity of microbial communities was decomposed into species replacement and richness differences. The results of the β‐diversity partition analysis showed that the differences in bacterial community composition in all areas were dominated by the species replacement process, contributing 64.1%, while the richness difference process contributed relatively little to the β‐diversity, contributing 17.9% (Figure 5b). Variation in fungal community composition across all areas was also dominated by species replacement processes, contributing 36.6%, while richness differences processes contributed a relatively small 17.8% to β‐diversity (Figure 5c). Thus, the variation in the microcosm system was mainly due to species replacement, with a relatively small contribution from richness differences (Figure 5a).

**FIGURE 6:**
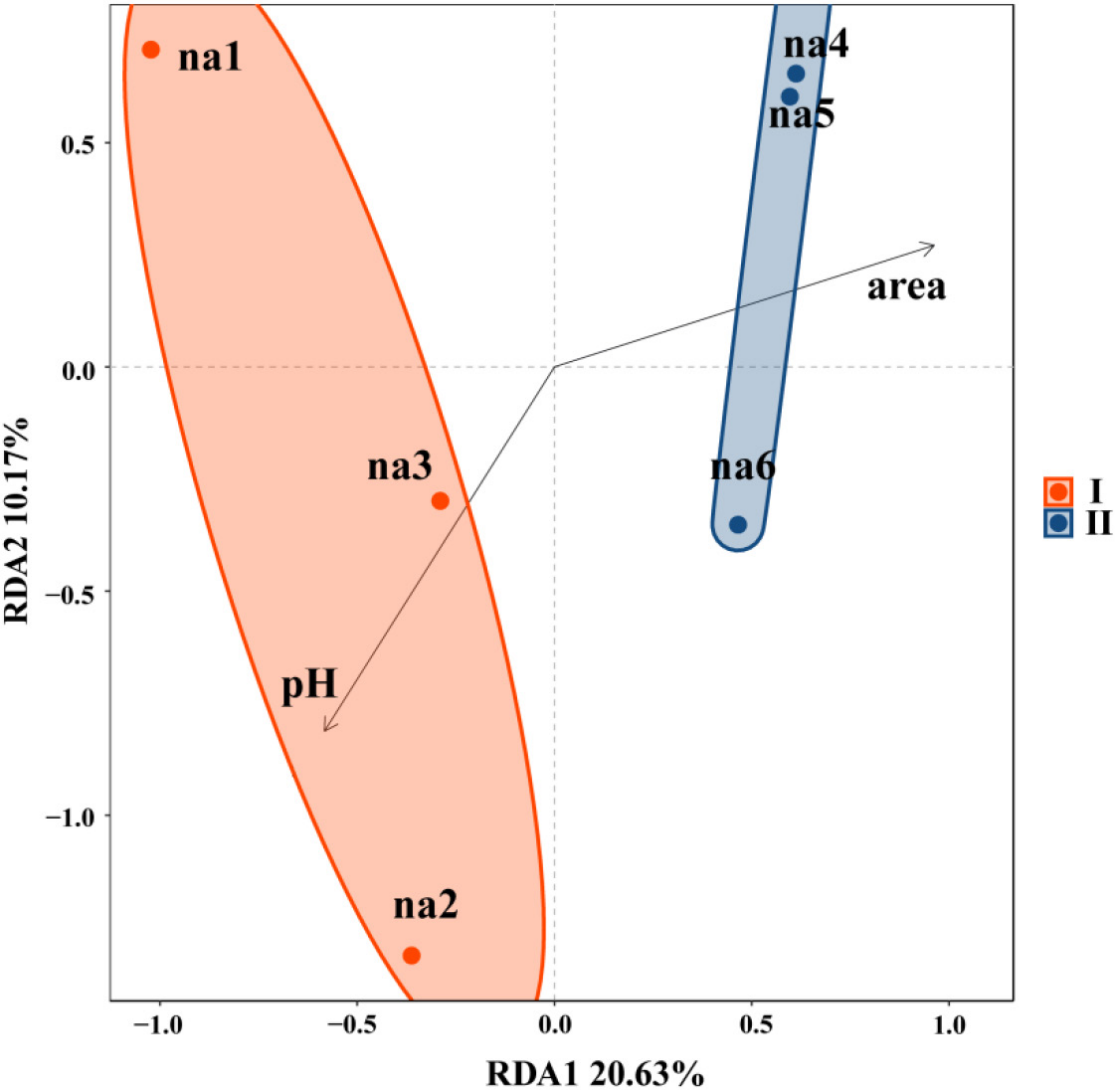
We using the na1‐6 represent the passive sampling areas from 0.25π to 50π cm^2^. Redundancy analysis Microcosms na1, na2, and na3 are the group I; microcosms na4, na5, and na6 are group II. Arrows indicate environmental factors and circles are 95% confidence intervals.

RDA was used to analyze species richness concerning environmental factors in a constrained manner, and the results showed that the two groups diverged. Open area contributed significantly to the second axis and dominated the between‐group variation. pH contributed significantly to the first axis and dominated the within‐group variation. However, the overall axis was less well explained at 30.8% (Figure 6).

## 4 DISCUSSION

The microcosm system used in this work ensured thorough sampling while higher‐resolution ASVs were used to estimate microbial diversity (Nearing et al., 2018). As the depth of the sequencing increases, the rarefaction curves rise and then quickly plateau, showing that microbial diversity monitoring is feasible, according to the rarefaction curves. We also made sure that the microorganisms only come from dispersal by using sterilized paocai soup. All of this makes sure that the sampling effect is eliminated, leaving only the dispersal effect. In this experimental system, however, there was no discernible relationship between species richness and the passive sampling area, and the dispersal effect had no impact on SAR. Any one of four explanations could account for this outcome.

Firstly, other ecological processes in the system obscured the shaping effect of dispersal on SAR after microbial dissemination. β‐diversity partition showed that species replacement was the main cause of community variations, with little contribution from species richness difference (Carvalho et al., 2013). The same number of biological niches were provided by the system in this experiment, and arriving bacteria had to occupy these finite spaces. The new species went in and only replaced the previous species once the ecological niches were filled, hence there was neither an increase nor a loss in the diversity of species. Furthermore, after microbial dispersal, extinction mechanisms are concealed (Niu et al., 2009; Vandermeer, 1972). Most common environmental microorganisms can grow in a neutral environment, and environmental microorganisms have varying degrees of tolerance to salt concentration and pH in paocai soup, with eventually tolerant species easily surviving the system and sensitive species frequently struggling to survive (Murphy et al., 2006). The paocai soup in this study shaped the microcosm, which has an extreme environment with a pH of 3.19–3.48 and high salinity. These findings indicate that, even though pH did not dominate the samples, high‐salinity and low‐pH microcosms have been selected for dispersal species. Simultaneously, in such a high‐stress environment with limited total environmental resources, the dominant *lactobacillus* produces acid, hydrogen peroxide, antibiotics, and other antagonistic substances that enhance interspecific interactions and exclude competition, exacerbating competitive relationships and interactions between microorganisms (Amarasekare & Nisbet, 2001; Chave et al., 2002; Deng et al., 2021; Hart et al., 2017; Muñoz‐Atienza et al., 2013). Species extinction occurs in the microcosm system due to both of these mechanisms of action. Perhaps the larger the area in the current study system has a higher rate of microbial dispersal, but the system's extinction process obscures this role. As a result, when there is only one effect of dispersal in the system, passive sampling may be able to shape significant SAR.

Secondly, microcosms with smaller passive sampling areas provide more space for reproduction at different temporal scales under the same environmental conditions, resulting in greater microbial richness in microcosms with smaller sampling areas. In this experiment, equal amounts of sterile paocai soup were used to establish a consistent microcosm background while keeping the microcosm size constant. Microorganisms entering the microcosm at different passive sampling sites face the same level of abiotic selection due to identical environmental conditions (Chisholm et al., 2016; Liu et al., 2020). Because there would be fewer species in limited areas, those that have adapted to the environment and survived will have greater space to reproduce. The number of species continues to grow with temporal scales.

Thirdly, the trade‐offs among speciation, extinction, and dispersal, three critical processes that change dynamically over temporal scales, impact the emphasis in island biogeography on species–area relationships (MacArthur & Wilson, 2016). Because of microorganisms' short life cycles, rapid community changes, and faster dynamics of their three fundamental processes, microbial SAR may not be constant over temporal scales. It also led us to observe no SAR.

Finally, the small island effect is expected to have an impact on SAR (Gao & Perry, 2016; Sfenthourakis et al., 2010; Triantis et al., 2012). The small island effect, which is consistent with the results of this experiment, is a phenomenon in which species richness is not significantly related to island size or grows at a slower rate than larger islands when island size falls below a certain threshold. Future research should include additional samples and island size gradients in their experimental design to further establish the significance of the small island effect.

If the SAR is still not present after excluding the above factors, then it indicates that the passive sampling hypothesis is just a pure sampling effect. In addition, there is some risk of chance that only one independent microcosm was set up for each area in this experiment. In future studies, we need to redesign a microcosm system that distinguishes microbial extinction from dispersal and explore the shaping of the passive sampling hypothesis on microbial SAR with a guaranteed sufficient sample size.

## AUTHOR CONTRIBUTIONS

**Wei Deng:** Conceptualization (equal); data curation (equal); formal analysis (equal); investigation (equal); methodology (equal); visualization (equal); writing – original draft (equal); writing – review and editing (equal). **Yiting Cheng:** Conceptualization (equal); data curation (equal); formal analysis (equal); investigation (equal); methodology (equal); visualization (equal); writing – original draft (equal); writing – review and editing (equal). **Zhengqiang Li:** Investigation (equal); resources (equal); writing – review and editing (supporting). **Faping Zhou:** Investigation (equal); resources (equal); writing – review and editing (supporting). **Xiaoyan yang:** Conceptualization (equal); funding acquisition (equal); investigation (equal); supervision (equal); writing – review and editing (equal). **Wen Xiao:** Conceptualization (equal); funding acquisition (equal); investigation (equal); project administration (lead); supervision (equal); writing – review and editing (equal).

## FUNDING INFORMATION

This work was funded by the Second Tibetan Plateau Scientific Expedition and Research Program (STEP), Grant No. 2019QZKK0402.

## CONFLICT OF INTEREST

The authors declare no competing or financial interests.

## Data Availability

Chinese Paocai microbiome ITS and 16S amplicon sequencing data are released (GSA: CRA008326, CRA008330). Please access it from the following link: https://bigd.big.ac.cn/gsa/browse/CRA008330 and https://bigd.big.ac.cn/gsa/browse/CRA008326.

## References

[ece39634-bib-0001] Adler, P. B. , & Lauenroth, W. K. (2003). The power of time: Spatiotemporal scaling of species diversity. Ecology Letters, 6(8), 749–756. 10.1046/j.1461-0248.2003.00497.x

[ece39634-bib-0002] Amarasekare, P. , & Nisbet, R. M. (2001). Spatial heterogeneity, source‐sink dynamics, and the local coexistence of competing species. The American Naturalist, 158, 572–584. 10.1086/323586 18707352

[ece39634-bib-0003] Azovsky, A. I. (2011). Species‐area and species‐sampling effort relationships: Disentangling the effects. Ecography, 34, 18–30. 10.1111/j.1600-0587.2010.06288.x

[ece39634-bib-0004] Ben‐Hur, E. , & Kadmon, R. (2020). Disentangling the mechanisms underlying the species‐area relationship: A mesocosm experiment with annual plants. Journal of Ecology, 108, 2376–2389. 10.1111/1365-2745.13476

[ece39634-bib-0005] Bidwell, M. T. , Green, A. J. , & Clark, R. G. (2014). Random placement models predict species‐area relationships in duck communities despite species aggregation. Oikos, 123, 1499–1508. 10.1111/oik.00821

[ece39634-bib-0006] Bolyen, E. , Rideout, J. R. , Dillon, M. R. , Bokulich, N. A. , Abnet, C. C. , Al‐Ghalith, G. A. , Alexander, H. , Alm, E. J. , Arumugam, M. , Asnicar, F. , Yang Bai, Y. , Bisanz, J. E. , Bittinger, K. , Brejnrod, A. , Brislawn, C. J. , Brown, C. T. , Callahan, B. J. , Caraballo‐Rodríguez, A. M. , Chase, J. , … Asnicar, F. (2019). Author correction: Reproducible, interactive, scalable and extensible microbiome data science using QIIME 2. Nature Biotechnology., 37, 1091. 10.1038/s41587-019-0252-6 PMC7015180 31341288

[ece39634-bib-0007] Bokulich, N. A. , Kaehler, B. D. , Rideout, J. R. , Dillon, M. , Bolyen, E. , Knight, R. , Huttley, G. A. , & Gregory Caporaso, J. (2018). Optimizing taxonomic classification of marker‐gene amplicon sequences with QIIME 2's q2‐feature‐classifier plugin. Microbiome., 6(1), 90. 10.1186/s40168-018-0470-z 29773078 PMC5956843

[ece39634-bib-0008] Bokulich, N. A. , Subramanian, S. , Faith, J. J. , Gevers, D. , Gordon, J. I. , Knight, R. , Mills, D. A. , & Caporaso, J. G. (2012). Quality filtering vastly improves diversity estimates from Illumina amplicon sequencing. Nature Methods, 10, 57–59. 10.1038/nmeth.2276 23202435 PMC3531572

[ece39634-bib-0009] Burns, K. C. , McHardy, R. P. , & Pledger, S. (2009). The small‐Island effect: Fact or artefact? Ecography, 32(2), 269–276. 10.1111/j.1600-0587.2008.05565.x

[ece39634-bib-0010] Callahan, B. J. , McMurdie, P. J. , Rosen, M. J. , Han, A. W. , Johnson, A. J. , & Holmes, S. P. (2016). DADA2: High‐resolution sample inference from Illumina amplicon data. Nature Methods, 13(7), 581–583. 10.1038/nmeth.3869 27214047 PMC4927377

[ece39634-bib-0011] Cam, E. , Nichols, J. D. , Hines, J. E. , Sauer, J. R. , Alpizar‐Jara, R. , & Flather, C. H. (2002). Disentangling sampling and ecological explanations underlying species‐area relationships. Ecology, 83(4), 1118–1130. 10.2307/3071918

[ece39634-bib-0012] Carvalho, J. C. , Cardoso, P. , Borges, P. A. V. , Schmera, D. , & Podani, J. (2013). Measuring fractions of β‐diversity and their relationships to nestedness: A theoretical and empirical comparison of novel approaches. Oikos, 122(6), 825–834. 10.1111/j.1600-0706.2012.20980.x

[ece39634-bib-0013] Chave, J. , Muller‐Landau, H. C. , & Levin, S. A. (2002). Comparing classical community models: Theoretical consequences for patterns of diversity. The American Naturalist, 159, 1–23. 10.1086/324112 18707398

[ece39634-bib-0014] Coleman, B. D. , Mares, M. A. , Willig, M. R. , & Hsieh, Y. H. (1982). Randomness, area, and species richness. Ecology, 63, 1121–1133. 10.2307/1937249

[ece39634-bib-0015] Connor, E. F. , & McCoy, E. D. (2013). Species‐area relationships. Encyclopedia of Biodiversity (Second Edition), 6, 397–411. https://10.1016/b978‐0‐12‐384719‐5.00132‐5

[ece39634-bib-0016] Connor, E. F. , & McCoy, E. D. M. (1979). The statistics and biology of the species‐area relationship. The American Naturalist, 113, 791–833. 10.1086/283438

[ece39634-bib-0017] Chisholm, R. A. , Fung, T. , Chimalakonda, D. , & O'Dwyer, J. P. (2016). Maintenance of biodiversity on islands. Proceedings of the Royal Society of London: Biological Sciences, 283, 0102. 10.1098/rspb.2016.0102 PMC4855381 27122558

[ece39634-bib-0018] Deng, W. , Yuan, C. L. , Li, N. , Liu, S. R. , Yang, X. Y. , & Xiao, W. (2021). Island formation history determines microbial species‐area relationships. Microbial Ecology. 10.1007/s00248-021-01906-5 34750668

[ece39634-bib-0019] Deng, W. , Liu, L. L. , Yu, G. B. , Li, N. , Yang, X. Y. , & Xiao, W. (2022). Testing the resource hypothesis of species‐area relationships: Extinction cannot work alone. Microorganisms, 10(10), 1993. 10.3390/microorganisms10101993 36296268 PMC9611600

[ece39634-bib-0020] DeSantis, T. Z. , Hugenholtz, P. , Larsen, N. , Rojas, M. , Brodie, E. L. , Keller, K. , Huber, T. , Dalevi, D. , Hu, P. , & Andersen, G. L. (2006). Greengenes, a chimera‐checked 16 S rRNA gene database and workbench compatible with ARB. Applied and Environmental Microbiology, 72, 5069–5072. 10.1128/AEM.03006-05 16820507 PMC1489311

[ece39634-bib-0021] Forester, B. R. , Lasky, J. R. , Wagner, H. H. , & Urban, D. L. (2018). Comparing methods for detecting multilocus adaptation with multivariate genotype‐environment associations. Molecular Ecology, 27, 2215–2233. 10.1111/mec.14584 29633402

[ece39634-bib-0022] Gao, D. , & Perry, G. (2016). Detecting the small Island effect and nestedness of herpetofauna of the West Indies. Ecology and Evolution, 6(15), 5390–5403. 10.1002/ece3.2289 27551391 PMC4984512

[ece39634-bib-0023] Gooriah, L. D. , & Chase, J. M. (2020). Sampling effects drive the species‐area relationship in lake zooplankton. Oikos, 129, 124–132. 10.1111/oik.06057

[ece39634-bib-0024] Gooriah, L. , Blowes, S. A. , Sagouis, A. , Schrader, J. , Karger, D. N. , Kreft, H. , & Chase, J. M. (2021). Synthesis reveals that Island species‐area relationships emerge from processes beyond passive sampling. Global Ecology and Biogeography, 30(10), 2119–2131. 10.1111/geb.13361

[ece39634-bib-0025] Guadagnin, D. L. , Maltchik, L. , & Fonseca, C. R. (2009). Species‐area relationship of neotropical waterbird assemblages in remnant wetlands: Looking at the mechanisms. Diversity and Distributions, 15(2), 319–327. 10.1111/j.1472-4642.2008.00533.x

[ece39634-bib-0026] Hart, S. P. , Usinowicz, J. , & Levine, J. M. (2017). The spatial scales of species coexistence. Nature Ecology & Evolution, 1, 1066–1073. 10.1038/s41559-017-0230-7 29046584

[ece39634-bib-0027] Hui, C. (2008). On species‐area and species accumulation curves: A comment on Chong and Stohlgren's index. Ecological Indicators, 8(3), 327–329. 10.1016/j.ecolind.2007.02.004

[ece39634-bib-0028] Kelly, B. J. , Wilson, J. B. , & Mark, A. F. (1989). Causes of the species‐area relation: A study of islands in Lake Manapouri, New Zealand. Journal of Ecology, 77(4), 1021–1028. 10.2307/2260820

[ece39634-bib-0029] Liu, J. , Matthews, T. J. , Zhong, L. , Liu, J. , Wu, D. , & Yu, M. (2020). Environmental filtering underpins the Island species‐area relationship in a subtropical anthropogenic archipelago. Journal of Ecology, 108, 424–432. 10.1111/1365-2745.13272

[ece39634-bib-0030] MacArthur, R. H. , & Wilson, E. O. (2016). The theory of Island biogeography. Princeton University Press. 10.1515/9781400881376

[ece39634-bib-0031] MacArthur, R. H. (1965). Patterns of species diversity. Biological Reviews, 40(4), 510–533. 10.1111/j.1469-185X.1965.tb00815.x

[ece39634-bib-0032] Marizzoni, M. , Gurry, T. , Provasi, S. , Greub, G. , & Cattaneo, A. (2020). Comparison of bioinformatics pipelines and operating Systems for the Analyses of 16 S rRNA gene amplicon sequences in human fecal samples. Frontiers in Microbiology, 11, 01262. 10.3389/fmicb.2020.01262 PMC7318847 32636817

[ece39634-bib-0033] Maryam, T. H. , & Ehsan, S. (2015). Molecular detection of endophytic, myrothecium spp. by its‐sequencing technique.

[ece39634-bib-0034] Muñoz‐Atienza, E. , Gómez‐Sala, B. , Araújo, C. , Campanero, C. , Campo, R. D. , Hernández, P. E. , Herranz, C. , & Cintas, L. M. (2013). Antimicrobial activity, antibiotic susceptibility and virulence factors of lactic acid bacteria of aquatic origin intended for use as probiotics in aquaculture. BMC Microbiology, 13(1), 15. 10.1186/1471-2180-13-15 23347637 PMC3574848

[ece39634-bib-0035] Manter, D. K. , & Vivanco, J. M. (2007). Use of the ITS primers, ITS1F and ITS4, to characterize fungal abundance and diversity in mixed‐template samples by QPCR and length heterogeneity analysis. Journal of microbiological methods, 71, 7–14. 17683818 10.1016/j.mimet.2007.06.016

[ece39634-bib-0036] Murphy, H. T. , Vanderwal, J. , Lovett‐Doust, L. , & Lowtt‐Doust, J. (2006). Invasiveness in exotic plants: immigration and naturalization in an ecological continuum. In M. W. Cadotte , S. M. McMahon , & T. Fukami (Eds.), Conceptual ecology and invasion biology: Reciprocal approaches to nature (pp. 65–105). Springer. 10.1007/1-4020-4925-0-4

[ece39634-bib-0037] Nearing, J. T. , Douglas, G. M. , Comeau, A. M. , & Langille, M. G. I. (2018). Denoising the denoisers: An independent evaluation of microbiome sequence error‐correction approaches. PeerJ, 6, e5364. 10.7717/peerj.5364 30123705 PMC6087418

[ece39634-bib-0038] Niu, K. , Liu, Y. , Shen, Z. , He, F. L. , & Fang, J. Y. (2009). Community assembly: The relative importance of neutral theory and niche theory. Biodiversity Science, 17(6), 579–593. 10.3724/SP.J.1003.2009.09142

[ece39634-bib-0039] Ohyama, L. , Holt, R. D. , Matthews, T. J. , & Lucky, A. (2021). The species‐area relationship in ant ecology. Journal of Biogeography, 48(8), 1824–1841. 10.1111/jbi.14149

[ece39634-bib-0040] Ouin, A. , Sarthou, J. P. , Bouyjou, B. , Deconchat, M. , Lacombe, J. P. , & Monteil, C. (2006). The species‐area relationship in the hoverfly (Diptera, Syrphidae) communities of Forest fragments in southern France. Ecography, 29(2), 183–190. 10.1111/j.2006.09067590.04135

[ece39634-bib-0041] Oksanen, J. , Blanchet, F. G. , Kindt, R. , Legendre, P. , Minchin, P. R. , O'Hara, R. B. , Simpson, G. L. , Sólymos, P. , Stevens, M. H. H. , & Wagner, H. (2012). Vegan: Community ecology package. Software http://CRAN.R‐project.org/package=vegan

[ece39634-bib-0042] Preston, F. W. (1960). Time and space and the variation of species. Ecology, 41(4), 611–627. 10.2307/1931793

[ece39634-bib-0043] Qian, W. , Chen, X. , Li, H. Q. , & Guo, S. (2021). Comparison of Illumina NovaSeq 6000 and MGISEQ‐2000 in profiling xenograft models. Cancer Research, 81, 259. 10.1158/1538-7445.AM2021-259

[ece39634-bib-0044] Ricklefs, R. E. , & Lovette, I. J. (1999). The roles of Island area per se and habitat diversity in the species‐area relationships of four lesser Antillean faunal groups. Journal of Animal Ecology, 68, 1142–1160. 10.1046/j.1365-2656.1999.00358.x

[ece39634-bib-0045] Rosenzweig, M. L. (1995). Species diversity in space and time. Cambridge University Press, 60(4), 971. 10.2307/3802400

[ece39634-bib-0046] Rosindell, J. , & Chisholm, R. A. (2021). The species‐area relationships of ecological neutral theory (pp. 259–288). Cambridge University Press. 10.1017/9781108569422.016

[ece39634-bib-0047] Sfenthourakis, S. , & Triantis, K. A. (2010). Habitat diversity, ecological requirements of species and the Small Island effect. Diversity & Distributions, 15(1), 131–140. 10.1111/j.1472-4642.2008.00526.x

[ece39634-bib-0048] Shen, C. , Gunina, A. , Luo, Y. , Wang, J. , He, J. Z. , Kuzyakov, Y. , Hemp, A. , Classen, A. T. , & Ge, Y. (2020). Contrasting patterns and drivers of soil bacterial and fungal diversity across a mountain gradient. Environmental Microbiology, 22(8), 3287–3301. 10.1111/1462-2920.15090 32436332

[ece39634-bib-0049] Storch, D. , Izling, A. L. , & Gaston, K. J. (2003). Geometry of the species‐area relationship in central European birds: Testing the mechanism. Journal of Animal Ecology, 72, 509–519. 10.1046/j.1365-2656.2003.00721.x

[ece39634-bib-0050] Tangney, R. S. , & Mark, W. (1990). Bryophyte Island biogeography: A study in Lake Manapouri, New Zealand. Oikos, 59(1), 21–26. 10.2307/3545117

[ece39634-bib-0051] Tjørve, E. , & Tjørve, K. M. (2017). Species‐area relationship. In John Wiley & Sons, Ltd (Ed.), eLS. John Wiley & Sons, Ltd. 10.1002/9780470015902.a0026330

[ece39634-bib-0052] Triantis, K. A. , & Sfenthourakis, S. (2012). Island biogeography is not a single‐variable discipline: The small Island effect debate. Diversity and Distributions, 18, 92–96. 10.1111/j.1472-4642.2011.00812.x

[ece39634-bib-0053] Vandermeer, J. H. (1972). Annual review of ecology and systematics. Niche theory, 3, 107–132. 10.1146/annurev.es.03.110172.000543

[ece39634-bib-0054] Wardle, D. A. (1999). Is "sampling effect" a problem for experiments investigating Biodiversit‐y‐ecosystem function relationships? Oikos, 87(2), 403–407. 10.2307/3546757

[ece39634-bib-0055] Wang, W. , Zhai, S. , Xia, Y. , Wang, H. , Ruan, D. , Zhou, T. , Zhu, Y. , Zhang, H. , Zhang, M. , Ye, H. , Ren, W. , & Yang, L. (2019). Ochratoxin A induces liver inflammation: Involvement of intestinal microbiota. Microbiome, 7(1), 151. 10.1186/s40168-019-0761-z 31779704 PMC6883682

[ece39634-bib-0056] White, T. J. , Bruns, T. D. , Lee, S. B. , & Taylor, J. W. (1990). Amplification and Direct Sequencing of Fungal Ribosomal RNA Genes for Phylogenetics. In M. A. Innis , D. H. Gelfand , J. J. Sninsky , & T. J. White (Eds.), PCR Protocols: A Guide to Methods and Applications (pp. 315–322). Academic Press. 10.1016/B978-0-12-372180-8.50042-1

[ece39634-bib-0057] Williams, C. B. (1943). Area and number of species. Nature, 152, 264–267. 10.1038/152264a0

[ece39634-bib-0058] Yamaura, Y. , Connor, E. F. , Royle, J. A. , Itoh, K. , Sato, K. , Taki, H. , & Mishima, Y. (2016). Estimating species‐area relationships by modeling abundance and frequency subject to incomplete sampling. Ecology and Evolution, 6(14), 4836–4848. 10.1002/ece3.2244 27547317 PMC4979711

